# World Health Organization recommendations on the treatment of drug-resistant tuberculosis, 2020 update

**DOI:** 10.1183/13993003.03300-2020

**Published:** 2021-06-03

**Authors:** Fuad Mirzayev, Kerri Viney, Nguyen Nhat Linh, Licé Gonzalez-Angulo, Medea Gegia, Ernesto Jaramillo, Matteo Zignol, Tereza Kasaeva

**Affiliations:** Global TB Programme, World Health Organization, Geneva, Switzerland

## Abstract

Antimicrobial resistance is a major public health problem globally. Likewise, forms of tuberculosis (TB) resistant to first- and second-line TB medicines present a major challenge for patients, healthcare workers and healthcare services. In November 2019, the World Health Organization (WHO) convened an independent international expert panel to review new evidence on the treatment of multidrug- (MDR) and rifampicin-resistant (RR) TB, using the Grading of Recommendations Assessment, Development and Evaluation approach.

Updated WHO guidelines emerging from this review, published in June 2020, recommend a shorter treatment regimen for patients with MDR/RR-TB not resistant to fluoroquinolones (of 9–11 months), with the inclusion of bedaquiline instead of an injectable agent, making the regimen all oral. For patients with MDR-TB and additional fluoroquinolone resistance, a regimen composed of bedaquiline, pretomanid and linezolid may be used under operational research conditions (6–9 months). Depending on the drug-resistance profile, extent of TB disease or disease severity, a longer (18–20 months) all-oral, individualised treatment regimen may be used. In addition, the review of new data in 2019 allowed the WHO to conclude that there are no major safety concerns on the use of bedaquiline for >6 months’ duration, the use of delamanid and bedaquiline together and the use of bedaquiline during pregnancy, although formal recommendations were not made on these topics.

The 2020 revision has highlighted the ongoing need for high-quality evidence and has reiterated the need for clinical trials and other research studies to contribute to the development of evidence-based policy.

## Abstract 2

**New WHO guidelines on the treatment of drug-resistant tuberculosis (TB) contain the latest recommendations on shorter or longer all oral treatment regimens for patients with drug-resistant TB, including the medicines to be used and other supportive measures** https://bit.ly/2UJeib7

## Introduction

Antimicrobial resistance is a major public health problem globally and has become a health security concern worldwide [1, 2]. Drug-resistant bacterial infections are on the rise globally, which has placed sharp focus on drug-resistant tuberculosis (TB), its diagnosis and treatment [3]. Over the past 20 years, it has become apparent that the widespread dissemination of drug-resistant TB will continue to challenge global efforts to cure patients and meet the ambitious targets of the End TB Strategy [4], the Sustainable Development Goals [5] and the targets arising from the Political Declaration of the United Nations High Level Meeting on TB [6].

Multidrug resistant (MDR)-TB is caused by *Mycobacterium tuberculosis* strains that are resistant to at least isoniazid and rifampicin, two first-line medicines used to treat TB [7]. The World Health Organization (WHO) estimates that 3.3% (95% CI 2.3–4.3%) of new and 18% (95% CI 9.7–27%) of previously treated TB cases occurring worldwide in 2019 had MDR/rifampicin-resistant (RR)-TB, which translates into close to half a million (range 400 000–535 000) new cases of MDR/RR-TB [8]. Globally, based on data reported by 105 countries and territories, the average proportion of MDR-TB patients with *M. tuberculosis* strains also resistant to one of the fluoroquinolones was 20.1% (95% CI 15.5–25.0%) [8]. Although countries have been expanding diagnostic capacity, detecting more patients with rifampicin resistance over recent years, only 206 030 patients (44% of the estimated total) were notified globally in 2019, indicating that drug susceptibility testing coverage is critically suboptimal [8]. Once diagnosed, the majority of identified MDR/RR-TB patients (86%) had access to treatment.

When compared to patients with drug susceptible disease, patients with MDR/RR-TB require treatment with regimens that are longer and have a higher potential for adverse events, depending on the second-line medications used. Over the past few decades, most MDR-TB regimens were designed to last ≥20 months [9]; however, shorter regimens (of 9–12 months’ duration) [10–16] have been recommended by the WHO since 2016 [17–19]. Despite this development, the programmatic implementation of both longer and shorter regimens continues to present challenges to patients and health systems due to the length and safety of these regimens, and the financial and social constraints associated with prolonged treatment and care [20–22].

Globally, national TB programmes reported to the WHO that only 57% of patients with MDR/RR-TB and 47% of those with MDR/RR-TB and resistance to fluoroquinolones had a successful treatment outcome based on the cohort who started treatment in 2017 [8]. While these figures have increased slightly in recent years, the rate of treatment success is still unacceptably low.

The development of normative guidance on TB treatment forms part of the WHO's core mandate to support national TB programmes in expanding access to care, in the context of the End TB Strategy [4, 23, 24]. Since 1996, the WHO Global TB Programme has regularly issued guidelines and implementation manuals for the treatment of drug-resistant TB [17, 19, 25–34]. Here, we describe the process and content of the latest policy update issued by the WHO in 2020 and discuss the implications of the changes for implementers involved in the clinical and programmatic management of drug-resistant TB [7]. Additional information on recommendations that existed prior to 2020 are not covered here, but are presented in [7].

## Process of guideline development

Overall, guideline development within the WHO is coordinated by technical programmes, ensuring that the process is evidence based and transparent, firmly relying on the highest ethical standards in science. The GRADE framework (Grading of Recommendations Assessment, Development and Evaluations) informs WHO guideline development processes [35, 36].

The update of the 2020 guidelines on drug-resistant TB treatment started in 2019 when the scope of the guideline update was defined, along with the research questions, in the Population, Intervention, Comparator, Outcomes (PICO) format (table 1). *Via* an online public comment platform, the WHO shared information on the goal and objectives of the guidelines update and the sources of new evidence that prompted the research questions, as well as the list of experts intending to participate in the guideline development group (GDG). Then, the WHO issued a public call, appealing to industry, researchers, national TB programmes and other agencies to provide suitable datasets that could add to the body of evidence related to the research questions. Ahead of the GDG meeting, the WHO commissioned systematic reviews and meta-analyses of available evidence on the effect of different treatment regimens on patient outcomes.

**TABLE 1:** Population, Intervention, Comparator, Outcome questions that were discussed during the guideline development group meeting on the treatment of drug-resistant tuberculosis (TB)

**Question 1**	In MDR/RR-TB patients, does an all-oral treatment regimen lasting 9–12 months and including bedaquiline safely improve outcomes when compared with other regimens conforming to WHO guidelines?
**Question 2**	In XDR-TB patients or patients who are treatment intolerant or with nonresponsive MDR-TB, does a treatment regimen lasting 6–9 months composed of bedaquiline, pretomanid and linezolid safely improve outcomes when compared with other regimens conforming to WHO guidelines?
**Question 3**	In MDR/RR-TB patients, does a treatment regimen containing bedaquiline for >6 months safely improve outcomes when compared with bedaquiline for ≤6 months as part of longer regimens otherwise conforming to WHO guidelines?
**Question 4**	In MDR/RR-TB patients, does concurrent use of bedaquiline and delamanid safely improve outcomes when compared with other treatment regimen options otherwise conforming to WHO guidelines?

MDR: multidrug-resistant; RR: rifampicin-resistant; XDR: extensively drug-resistant; WHO: World Health Organization.

An international team of experts with broad technical knowledge of drug-resistant TB and carefully vetted for any conflicts of interest formed a GDG. All conflicts of interest and their management are published in the annexes of the guidelines [36, 37]. The membership of the GDG represented people from varied geographic and health systems settings, future users of the guidelines as well as community and patient representatives. A number of online webinars were held with the GDG before the face-to-face meeting was held in Geneva.

## The scope of the updated guidelines

The scope of the 2020 guidelines update covered four areas, as follows. 1) The effectiveness and safety of a standardised all-oral bedaquiline-containing regimen lasting 9–12 months for the treatment of patients with MDR/RR-TB; 2) the effectiveness and safety of a 6–9-month regimen composed of bedaquiline, pretomanid and linezolid (BPaL) for the treatment of patients with MDR-TB and additional fluoroquinolone resistance; 3) the use of bedaquiline as part of longer regimens for >6 months; and 4) the concurrent use of bedaquiline and delamanid as part of longer regimens. In addition, new evidence on the use of bedaquiline during pregnancy complemented the evidence being assessed for the four PICO questions. All relevant outcomes for these PICO questions were scored in the critical range by the GDG members, prior to the GDG face-to-face meeting (table 2). The scope of the 2020 guidelines update did not include aspects of the programmatic management of drug-resistant TB for which no substantive new evidence had emerged since the previous version of the guidelines published in 2019 [7].

**TABLE 2:** Scoring of outcomes considered relevant by the World Health Organization convened guideline development group for the evidence review for the 2020 update on multidrug/rifampicin-resistant tuberculosis (TB) treatment

**Outcomes (as outlined in the scoping proposal)**	**Rating**
**Survival (or death)**	8.33
**Relapse-free cure**	8.22
**Bacteriological cure by end of treatment**	8.19
**Successful completion of treatment (or lack of successful completion)**	7.96
**Treatment failure or relapse**	7.93
**Adherence to treatment (or treatment interruption due to nonadherence)**	7.48
**Acquisition (amplification) of drug resistance**	7.33
**Adverse events from anti-TB medicines**	7.19

Relative importance was rated on an incremental scale, as follows. 1–3 points: not important for making recommendations; 4–6 points: important, but not critical for making recommendations; 7–9 points: critical for making recommendations on the evaluated interventions.

## Analysis and review of evidence

Detailed statistical analysis plans were prepared for the analytical approach to each PICO question. Descriptive analyses were performed to determine population characteristics and to provide information on the variables needed for matching and adjustment. Then, a combination of exact matching and propensity score-based matching on several variables (covariates) was used to minimise bias and confounding. The distribution of the matched covariates within the intervention and comparator groups was assessed to determine the fidelity of the matching process. Logistic regression and binomial regression were used to calculate absolute and relative estimates of effect and their 95% confidence intervals, based on comparisons of pooled data from the included studies and datasets.

Based on the results of the analyses, GRADE evidence profiles were prepared for each PICO question or sub-question using the GRADEpro online application (www.gradepro.org). Also in the GRADE evidence profile, the quality of the evidence was assessed using the following criteria: study design, risk of bias, imprecision, inconsistency, indirectness, publication bias, magnitude of effect, plausible confounding and the dose–effect response gradient [38]. GRADE evidence profiles were discussed by the GDG before the development of “evidence to decision (EtD)” frameworks. These frameworks captured the judgements of the GDG in selected areas that relate to the PICO questions and the evidence presented, including but not limited to the desirable and undesirable effects of the intervention; the balance of these effects; the certainty of the evidence; the effect of the intervention on resource use, equity, feasibility and acceptability; and the value that people would place on the outcomes of interest. In addition, the EtD frameworks captured a narrative summary of the content of the GDG discussions, with explanatory remarks on the GDG's judgements as well as a description of the wording and strength of each recommendation and any accompanying remarks [7]. The GRADE evidence tables and EtD frameworks are available in their entirety in the guideline annexes [37].

## Summary of evidence and analyses

### PICO question: in MDR/RR-TB patients, does an all-oral treatment regimen lasting 9–12 months and including bedaquiline safely improve outcomes when compared with other regimens conforming to WHO guidelines?

The evidence reviewed on the shorter all-oral bedaquiline-containing regimen was derived from programmatic data from South Africa's Electronic Drug-Resistant Tuberculosis Register (www.edrweb.net). 10 152 records of patients with MDR/RR-TB initiating TB treatment anytime between January and June 2017 were available, of which 891 patients who received an all-oral bedaquiline-containing shorter regimen were included in the primary analyses. These data were compared to 987 patients treated with a shorter regimen which included an injectable agent; 1437 patients treated with longer regimens that conformed to previous WHO recommendations in the 2016 guidelines update [19]; and 474 patients treated with longer regimens that included bedaquiline in combination with other medicines. The comparison data were derived from an individual patient dataset (IPD) which comprises 13 273 individual patient records from 55 different studies/centres in 38 countries.

The analysis indicated that the use of an all-oral bedaquiline-containing shorter regimen in patients with MDR/RR-TB was associated with higher treatment success rates (73% *versus* 60% when compared to the group who received a standardised shorter regimen with an injectable included). Adjusted odds ratios (aOR) for this comparison were 2.1 (95% CI 1.1–4.0) for the treatment outcomes of success *versus* failure/recurrence combined; 1.6 (95% CI 1.2–2.1) for success *versus* death; 1.7 (95% CI 1.3–2.2) for success *versus* failure/recurrence and death combined; and 1.9 (95% CI 1.6–2.4) for success *versus* all unfavourable outcomes combined (*i.e.* failure/relapse/death and loss to follow-up combined). Rates of loss to follow-up were lower among the group who received bedaquiline as part of the shorter regimen when compared to those who received the shorter regimen which contained an injectable agent (9.9% *versus* 17.3%; aOR 0.5, 95% CI 0.4–0.7).

### PICO question: in extensively drug-resistant TB patients or patients who are treatment intolerant or with nonresponsive MDR-TB, does a treatment regimen lasting 6–9 months composed of bedaquiline, pretomanid and linezolid safely improve outcomes when compared with other regimens conforming to WHO guidelines?

The evidence to inform this PICO question was derived from the Nix-TB study [39] conducted by TB Alliance, where the total study population was 109 patients. One patient withdrew informed consent to participate in the study and this person was included in safety analyses, but not in the analyses for effectiveness. These data were compared to a subset of data from the IPD. For the primary analyses, the comparator group included patients from the IPD on longer treatment regimens (with a mean duration of treatment of 21.0–25.5 months), who received both bedaquiline and linezolid as part of the regimen (no patients received pretomanid in the IPD). This comparison group included data from 456 patients reported in studies, including patients from Belarus, India, France, Russia, China, South Korea, the Philippines, Thailand, Russia and South Africa.

Overall, when comparing treatment success *versus* failure or recurrence, the treatment success rate in the Nix-TB study was 97.0% compared to 91.7% in the comparator group. For the comparison of treatment success *versus* death, treatment success was 93.2% in the Nix-TB study compared to 91.9% in the comparator group. For the comparisons of treatment success *versus* failure or recurrence or death and treatment success *versus* all unfavourable outcomes, the proportions of patients with treatment success in the intervention and comparator groups were 90.5% *versus* 84.8%, and 88.9% *versus* 82.2%, respectively. Based on these figures, the primary analysis yielded aORs of 3.3 for treatment success *versus* failure or recurrence (95% CI 0.8–13.7); 1.0 for success *versus* death (95% CI 0.1–8.2); 1.8 for success *versus* failure or relapse or death (95% CI 0.7–4.4); and 1.2 for success *versus* all unfavourable outcomes (95% CI 0.5–3.1). The proportion of patients who were lost to follow-up was lower in the intervention (BPaL) group (1.8%) compared to the comparison group (3.1%).

With regards to adverse events, of the 109 patients in the Nix-TB study, 28 (25.7%) experienced at least one serious adverse event. This included one death related to acute haemorrhagic pancreatitis (0.9%), 29 (26.6%) other serious adverse events including hospitalisations and life-threatening events and two (1.8%) adverse events that resulted in persistent or significant disability or incapacity. A total of 53 (49%) patients experienced at least one grade 3–4 adverse event considered to be related to the study drugs; these comprised 25 with peripheral neuropathy (11 resolved), 16 with increased hepatic transaminases (13 resolved), nine with haematological adverse events (all resolved), eight with increased pancreatic enzymes (seven resolved) and two with optic neuritis (both resolved). Information from the independent review on the pre-clinical and early-phase clinical data described that safety signals were observed at exposures that are higher than would be used in humans; however, safety signals of note included liver toxicities (hypertrophy of hepatocytes, transaminase elevation and increased liver weight, observed at higher doses in rodents and lower doses in monkeys) and reproductive toxicities in males, observed in animal (murine and simian) models, which appear to be both time and dose dependent.

### PICO question: in MDR/RR-TB patients, does a treatment regimen containing bedaquiline for >6 months safely improve outcomes when compared with bedaquiline for >6 months as part of longer regimens otherwise conforming to WHO guidelines?

For this PICO question the data were derived from the EndTB observational study [40], with the overall dataset comprising a total of 1094 patients from 13 countries [40]. These countries are Armenia, Bangladesh, Belarus, Democratic People's Republic of Korea, Ethiopia, Georgia, Indonesia, Kazakhstan, Kenya, Lesotho, Myanmar, Pakistan and Peru. Of the 515 records which met the inclusion criteria, 242 patients who received bedaquiline for >203 days in total comprised the intervention group. They were compared to 273 patients from the EndTB observational study who received bedaquiline for a total of between 168 and 203 days. 203 days was chosen as a cut-off as the intermodal trough of bedaquiline use for all patients in the EndTB observational study was 203 days. It should be noted that the cut-off was not 6 months exactly, but 203 days.

The analysis yielded aORs of 1.5 (95% CI 0.7–2.7) for treatment success *versus* failure; 0.8 (95% CI 0.2–0.4) for treatment success *versus* death; 1.0 (95% CI 0.5–1.7) for treatment success *versus* failure/death; and 0.8 (95% CI 0.5–1.2) for treatment success *versus* all unfavourable outcomes. Due to an inability to apply the pre-defined analytical methods, there was a high likelihood of residual confounding in the data used to assess the PICO question about the use of bedaquiline for >6 months. The available data from the patient population in the study did not permit extrapolation to routine use in all MDR/RR-TB patients. This precluded a formal recommendation on the efficacy or effectiveness of bedaquiline use beyond 6 months’ duration. However, the evidence supports the safe use of bedaquiline beyond 6 months in patients who receive appropriate schedules of baseline and follow-up monitoring and management of adverse drug reactions.

### PICO question: in MDR/RR-TB patients, does concurrent use of bedaquiline and delamanid safely improve outcomes when compared with other treatment regimen options otherwise conforming to WHO guidelines?

For this final PICO question, the data were derived from the same cohort of patients in the EndTB observational study [41] that informed the PICO question on bedaquiline use beyond 6 months’ duration. However, in this dataset only 92 patients received both medicines together for any period of time and even fewer started bedaquiline and delamanid at the same time and by the end of the first month of treatment (n=38). An additional data source comprised a cohort of 100 patients treated with bedaquiline in Mumbai, India (from a Médecins Sans Frontières-supported project). The total intervention population therefore comprised 84 patients; 38 from the EndTB observational study cohort and 46 from the dataset from Mumbai. The primary comparison group included 401 patients (n=302 from the EndTB observational study), and 99 patients from two other datasets (n=82 from a dataset provided by the Republic of Belarus and data on another 17 patients shared by the Republic of Uzbekistan).

The analyses yielded aORs of 1.6 (95% CI 0.5–5.4) for treatment success *versus* treatment failure; 0.8 (95% CI 0.3–2.1) for treatment success *versus* death; 1.2 (95% CI 0.6–2.5) for treatment success *versus* failure/death; and 0.6 (95% CI 0.3–1.1) for treatment success *versus* all unfavourable outcomes. With regards to adverse events, among the 92 patients receiving bedaquiline with concomitant delamanid during treatment in the EndTB observational study (total exposure of 1095 person-months), two bedaquiline-related adverse events and three delamanid-related adverse events occurred (combined rate 0.46 per 100 person-months of exposure, with two of these five events classified as serious). This rate was comparable to the rates among people receiving bedaquiline alone and delamanid alone (0.41 and 0.68 per 100 person-months of exposure, respectively). No fatal drug-related adverse events occurred among patients receiving bedaquiline and delamanid concurrently and there was one person who experienced a grade 3 prolongation of QT interval corrected using Fridericia's formula (QTcF).

Additional data presented included safety data from the Delamanid Bedaquiline for Resistant Tuberculosis (DELIBERATE) trial (AIDS Clinical Trials Group A5343) [42]. In this randomised controlled trial, among the patients randomised to bedaquiline (n=28), delamanid (n=27) or both medicines (n=27), the on-treatment change in QTcF from baseline was 11.9 ms, 8.6 ms and 20.7 ms, respectively [42]. Of the 27 patients who received both medicines, 10 (37.0%) experienced a grade 1 QT adverse event_,_ while two (7.4%) experienced a grade 2 QT adverse event, comparable to patients in the bedaquiline arm and the delamanid (32.0% and 41.0% of patients experienced a grade 1 QT adverse event and 3.6% and 7.4% of patients experienced a grade 2 QT adverse event, respectively). In the DELIBERATE trial a grade 1 QT adverse event was classified as an absolute QTcF >480 ms and ≤500 ms and QTcF change from baseline from >0 ms and ≤30 ms or an absolute QTcF ≤480 ms and QTcF change from baseline from >30 ms and ≤60 ms. A grade 2 QT adverse event was classified as an absolute QTcF >480 ms and ≤500 ms and QTcF change from baseline from >30 ms and ≤60 ms or an absolute QTcF ≤480 ms and QTcF change from baseline >60 ms. A grade 3 QT adverse event was classified as an absolute QTcF >500 ms or an absolute QTcF >480 ms and QTcF change from baseline >60 ms, whereas a grade 4 QT adverse event was a life-threatening consequence, for example torsade de pointes or other associated serious ventricular dysrhythmia [42]. QTcF prolongation >500 ms was rare, occurring in only one patient, and no patients experienced grade 3 or 4 QT adverse events [42].

There was insufficient evidence to assess the efficacy or effectiveness of concomitant use of bedaquiline and delamanid. The data did not lend to a meaningful analysis for the secondary comparator (extended use of delamanid alone) as the populations were too different to allow for the matching that is usually carried out. This precluded a formal recommendation on the efficacy or effectiveness of concomitant use of bedaquiline and delamanid. However, the data suggested no additional safety concerns with regards to the concurrent use of bedaquiline and delamanid. Therefore, bedaquiline and delamanid may be concurrently used in patients who have limited other treatment options, *i.e.* for patients with a small number of other effective drugs included in their regimen, probably due to an extensive drug-resistance profile or intolerance to other second-line TB medications. Appropriate schedules of safety monitoring (at baseline and throughout treatment) should be in place for these patients, including ECG and electrolyte monitoring, and clinicians should be cognisant of other medicines in the regimen that have the potential to either prolong the QT interval or cause other potential adverse events.

### Additional operational issue: bedaquiline use during pregnancy

Additional data were reviewed from a South African cohort study on the use of bedaquiline during pregnancy [43]. This study included information from 108 pregnant women with RR-TB who were recruited from one MDR/RR-TB referral hospital in South Africa between January 2013 and December 2017 [43]. 58 women received bedaquiline as part of their MDR/RR-TB regimen and were compared to 50 women who had no bedaquiline in their regimen. The women in this study gave birth to 109 live infants of whom 49 had bedaquiline exposure *in utero* and 60 had no bedaquiline exposure *in utero*.

With regards to bedaquiline exposure during pregnancy, the findings of the cohort study demonstrated no statistically significant differences in birth or pregnancy outcomes when comparing infants who had intrauterine bedaquiline exposure *versus* those who did not have this exposure (p=0.741 for birth outcomes and p=0.312 for pregnancy outcomes) [43]. Of all pregnancy and infant outcomes assessed, only low birthweight was associated with bedaquiline exposure *in utero* (45% *versus* 26%, p=0.034). However, it was not possible to conclusively ascribe this effect to bedaquiline, and more investigation is needed to explore this relationship [43]. There were no significant differences in infant growth after birth (in a subanalysis of 86 babies followed-up prospectively (41 exposed to bedaquiline *in utero* and 45 not exposed) [43]. Additionally, treatment outcomes were favourable for pregnant women exposed to bedaquiline *versus* not those exposed (treatment success 71% *versus* 62%, respectively, p=0.349) [43].

Amikacin, streptomycin, prothionamide and ethionamide are usually contraindicated during pregnancy. Based on the new evidence provided from South Africa, fetal exposure to bedaquiline *in utero* was associated with low birthweight [43]. However, there were no other significant differences in infant outcomes, pregnancy outcomes or maternal treatment outcomes, including weight gain in the infants until 1 year of age [43]. It is recommended that in such cases, a longer regimen be individualised to include components with a safety profile that has already been well established. The outcomes of treatment and pregnancy, including data from post-partum surveillance for congenital anomalies should be documented to help inform future recommendations for MDR-TB treatment during pregnancy.

### Recommendations and remarks

The grouping of medicines used for the treatment of MDR/RR-TB and extensively drug-resistant (XDR)-TB as part of longer regimens was previously revised based on the GDG discussions in 2018, and this grouping of medicines remains the same in the current update [7]. These medicines and their grouping are provided in table 3 [7]. The WHO considers that only the medicines in this table are relevant to MDR/RR-TB treatment regimens under programmatic conditions. The new recommendations for the shorter all-oral bedaquiline-containing regimen for MDR/RR-TB and for the BPaL regimen for MDR-TB with additional fluoroquinolone resistance are provided below. Other recommendations about the medicines to be used in longer regimens, the duration and monitoring of longer regimens, the use of surgery for MDR/RR-TB, the use of antiretrovirals during TB treatment for people living with HIV infection, and patient support remain unchanged and are reproduced in the updated guidelines released by the WHO in 2020, so that all WHO recommendations for the treatment of MDR/RR-TB are in one document [7].

**TABLE 3:** Grouping of medicines recommended for use in longer multidrug-resistant (MDR) tuberculosis (TB) regimens

	**Steps**	**Medicine**	**Abbreviation**
**Group A**	Include all three medicines	Levofloxacin OR moxifloxacin	Lfx Mfx
		Bedaquiline^#,¶^	Bdq
		Linezolid^+^	Lzd
**Group B**	Add one or both medicines	Clofazimine	Cfz
		Cycloserine OR terizidone	Cs Trd
**Group C**	Add to complete the regimen and when medicines from groups A and B cannot be used	Ethambutol	E
		Delamanid^¶^^,^^§^	Dlm
		Pyrazinamide^ƒ^	Z
		Imipenem–cilastatin OR meropenem^##^	Ipm–Cln Mpm
		Amikacin (OR streptomycin)^¶¶^	Am (S)
		Ethionamide OR prothionamide^++^	Eto Pto
		p-Aminosalicylic acid^++^	PAS

This table is intended to guide the design of individualised longer MDR-TB regimens (the composition of the recommended shorter MDR-TB regimen is largely standardised; see Section 2 on shorter all-oral bedaquiline-containing regimen for multidrug- or rifampin-resistant TB in the guidelines). Medicines in group C are ranked by decreasing order of usual preference for use subject to other considerations. The 2018 individual patient dataset (IPD) meta-analysis for longer regimens included no patients on thioacetazone and too few patients on gatifloxacin and high-dose isoniazid for a meaningful analysis. No recommendation on perchlozone, interferon-γ or sutezolid was possible owing to the absence of final patient treatment outcome data from appropriate studies. ^#^: bedaquiline is usually administered 400 mg orally once daily for the first 2 weeks, followed by 200 mg orally three times per week for 22 weeks (total duration of 24 weeks). Evidence on the safety and effectiveness of bedaquiline use beyond 6 months and below the age of 6 years was insufficient for review in 2018. Therefore, the use of bedaquiline beyond 6 months was implemented following best practices in “off-label” use [46]. New evidence on the safety profile of bedaquiline use beyond 6 months was available to the guideline development group (GDG) in 2019. Based on this evidence, the GDG were not able to assess the impact of prolonged bedaquiline use on efficacy, due to the limited evidence and potential residual confounding in the data. However, the evidence supports the safe use of bedaquiline beyond 6 months in patients who receive appropriate schedules of baseline and follow-up monitoring. It is important to note that the use of bedaquline beyond 6 months still remains as off-label use and in this regard best practices in off-label use still apply. ^¶^: evidence on the concurrent use of bedaquiline and delamanid was insufficient for review in 2018. In 2019, new evidence on the concurrent use of bedaquiline and delamanid was made available to the GDG. With regards to safety, the GDG concluded that the data suggest no additional safety concerns with regards to concurrent use of bedaquiline and delamanid. Both medicines may be used concurrently among patients who have limited other treatment options available to them, and if sufficient monitoring (including baseline and follow-up ECG and electrolyte monitoring) is in place. The data on the effectiveness of concurrent use of bedaquiline and delamanid were reviewed by the GDG, but due to the limited evidence and potential residual confounding in the data, the GDG were unable to proceed with a recommendation on effectiveness. ^+^: use of linezolid for ≥6 months was shown to increase effectiveness, although toxicity may limit use. The analysis suggested that using linezolid for the whole duration of treatment would optimise its effect (∼70% of patients on linezolid with data received it for >6 months and ∼30% for 18 months or the whole duration). No patient predictors for early cessation of linezolid could be inferred from the IPD sub-analysis. ^§^: evidence on the safety and effectiveness of delamanid beyond 6 months and below the age of 3 years was insufficient for review. Use of delamanid beyond these limits should follow best practices in “off-label” use [30]. ^ƒ^: pyrazinamide is counted as an effective agent only when drug susceptibility test (DST) results confirm susceptibility. ^##^: every dose of imipenem–cilastatin and meropenem is administered with clavulanic acid, which is available only in formulations combined with amoxicillin. Amoxicillin–clavulanic acid is not counted as an additional effective TB agent and should not be used without imipenem–cilastatin or meropenem. ^¶¶^: amikacin and streptomycin are to be considered only if DST results confirm susceptibility and high-quality audiometry monitoring for hearing loss can be ensured. Streptomycin is to be considered only if amikacin cannot be used (unavailable or documented resistance) and if DST results confirm susceptibility (resistance to streptomycin is not detectable with second-line molecular line probe assays and phenotypic DST is required). Kanamycin and capreomycin are no longer recommended for use in MDR-TB regimens. ^++^: these agents showed effectiveness only in regimens without bedaquiline, linezolid, clofazimine or delamanid, and are thus proposed only when other options to compose a regimen are not possible.

The certainty of the evidence reviewed in 2019 to address the PICO questions on the shorter all-oral bedaquiline-containing regimen and the BPaL regimen was rated as very low and the two new recommendations proposed by the GDG were both graded as conditional (table 4). There were no formal recommendations made on the use of bedaquiline >6 months duration, the use of bedaquiline and delamanid together or the use of bedaquiline during pregnancy; however, the GDG were able to make some statements about their safe use.

**TABLE 4:** Perspective taken and description of strength and conditionality of recommendations

	**Strong recommendation**	**Conditional recommendation**
**Patients**	Most individuals in this situation would want the recommended course of action and only a small proportion would not Formal decision aids are not likely to be needed to help individuals make decisions consistent with their values and preferences	The majority of individuals in this situation would want the suggested course of action, but many would not
**Clinicians**	Most individuals should receive the intervention Adherence to this recommendation according to the guidelines could be used as a quality criterion or performance indicator	Recognise that different choices will be appropriate for individual patients, and that patients must be helped to arrive at a management decision consistent with their values and preferences Decision aids may be useful in helping individuals to make decisions consistent with their values and preferences
**Policy-makers**	The recommendation can be adopted as policy in most situations	Policy-making will require substantial debate and involvement of various stakeholders

#### Shorter all-oral bedaquiline-containing regimen for MDR/RR-TB

A shorter, all-oral, bedaquiline-containing regimen of 9–12 months duration is recommended in eligible patients with confirmed MDR/RR-TB who have not been exposed to treatment with second-line TB medicines used in this regimen for >1 month and in whom resistance to fluoroquinolones has been excluded (conditional recommendation, very low certainty in the evidence).

The evidence review for this recommendation focused on the shorter regimen where the injectable agent was replaced by bedaquiline (used for 6 months), in combination with levofloxacin or moxifloxacin, ethionamide, ethambutol, isoniazid (high dose), pyrazinamide and clofazimine for 4 months (with the possibility to extend to 6 months if the patient remained sputum smear-positive at the end of 4 months); followed by 5 months of treatment with levofloxacin or moxifloxacin, clofazimine, ethambutol and pyrazinamide. After taking into account patient preference and clinical judgement, this regimen can be a preferred option for patients in whom all of the following apply: confirmed MDR/RR-TB (with at least confirmed resistance to rifampicin), with resistance to fluoroquinolones ruled out, exposure to previous treatment with second-line medicines for no more than 1 month, no extensive TB disease and no severe extrapulmonary TB. Extensive (or advanced) TB disease is defined as the presence of bilateral cavitary disease or extensive parenchymal damage on chest radiography. In children aged <15 years, advanced disease is usually defined by the presence of cavities or bilateral disease on chest radiography. Severe extrapulmonary TB is defined as the presence of miliary TB or TB meningitis. In children aged <15 years, extrapulmonary forms of disease other than lymphadenopathy (peripheral nodes or isolated mediastinal mass without compression) are considered as severe (adapted from [44]). The evidence reviewed supports the use of this regimen in patient subgroups, such as people living with HIV (see sub-group considerations in [7]). Implementation of this regimen requires access to perform rapid drug-susceptibility testing (DST) against fluoroquinolones.

#### The BPaL regimen for MDR-TB with additional fluoroquinolone resistance

A treatment regimen lasting 6–9 months composed of bedaquiline, pretomanid and linezolid (BPaL) may be used under operational research conditions in MDR-TB patients with TB that is resistant to fluoroquinolones who have had no previous exposure to bedaquiline and linezolid for >2 weeks (conditional recommendation, very low certainty in the estimates of effect).

The BPaL regimen showed high rates of treatment success when used in XDR-TB patients in South Africa and notable additional favourable treatment outcomes resulting from the use of the regimen when compared to patients receiving longer regimens with bedaquiline and linezolid. However, there were important residual concerns about the likelihood and severity of adverse events, possible reproductive toxicity signals in the pre-clinical data, limitations in the study design and the overall very low certainty of the evidence.

The BPaL regimen (used in the Nix-TB study) [39] may not be considered for routine programmatic use worldwide until additional evidence on efficacy and safety has been generated. However, in individual patients for whom the design of an effective regimen based on existing WHO recommendations is not possible, the BPaL regimen may be considered as a last resort under prevailing ethical standards.

The evidence reviewed supports the use of this regimen in certain patient sub-groups such as people living with HIV (see sub-group considerations in [7]).

A summary of all recommendations included in the *WHO Consolidated Guidelines on Tuberculosis, Module 4: Drug-Resistant Tuberculosis Treatment* highlighting changes when compared to the previous guideline, is included in table 5.

**TABLE 5:** Summary of changes to the World Health Organization (WHO) multidrug-resistant (MDR)/rifampicin-resistant (RR) tuberculosis (TB) treatment recommendations between 2019 and current updates

**Recommendations in the 2019 update**	**Recommendations in the current update**
**Section 1: Regimens for isoniazid-resistant TB**	**Section 1: Regimen for rifampicin-susceptible and isoniazid-resistant TB**
In patients with confirmed rifampicin-susceptible and isoniazid-resistant TB, treatment with rifampicin, ethambutol, pyrazinamide and levofloxacin is recommended for a duration of 6 months (Conditional recommendation, very low certainty in the estimates of effect)	1.1 In patients with confirmed rifampicin-susceptible, isoniazid-resistant TB (Hr-TB), treatment with rifampicin, ethambutol, pyrazinamide and levofloxacin is recommended for a duration of 6 months (Conditional recommendation, very low certainty in the estimates of effect) (No change)
In patients with confirmed rifampicin-susceptible and isoniazid-resistant TB, it is not recommended to add streptomycin or other injectable agents to the treatment regimen (Conditional recommendation, very low certainty in the estimates of effect)	1.2 In patients with confirmed rifampicin-susceptible, isoniazid-resistant TB, it is not recommended to add streptomycin or other injectable agents to the treatment regimen (Conditional recommendation, very low certainty in the estimates of effect) (No change)
**Section 2: The composition of longer MDR-TB regimens**	**Section 3: Longer regimens for MDR/RR-TB**
In MDR/RR-TB patients on longer regimens, all three group A agents and at least one group B agent should be included to ensure that treatment starts with at least four TB agents likely to be effective, and that at least three agents are included for the rest of the treatment after bedaquiline is stopped.^#^ If only one or two group A agents are used, both group B agents are to be included. If the regimen cannot be composed with agents from groups A and B alone, group C agents are added to complete it (Conditional recommendation, very low certainty in the estimates of effect)	3.1 In MDR- or RR-TB (MDR/RR-TB) patients on longer regimens, all three group A agents and at least one group B agent should be included to ensure that treatment starts with at least four TB agents likely to be effective, and that at least three agents are included for the rest of treatment if bedaquiline is stopped. If only one or two group A agents are used, both group B agents are to be included. If the regimen cannot be composed with agents from groups A and B alone, group C agents are added to complete it (Conditional recommendation, very low certainty in the estimates of effect) (Editing of the word “after” to “if” with reference to stopping bedaquiline)
Kanamycin and capreomycin are not to be included in the treatment of MDR/RR-TB patients on longer regimens (Conditional recommendation, very low certainty in the estimates of effect)	3.2 Kanamycin and capreomycin are not to be included in the treatment of MDR/RR-TB patients on longer regimens (Conditional recommendation, very low certainty in the estimates of effect) (No change)
Levofloxacin or moxifloxacin should be included in the treatment of MDR/RR-TB patients on longer regimens (Strong recommendation, moderate certainty in the estimates of effect)	3.3 Levofloxacin or moxifloxacin should be included in the treatment of MDR/RR-TB patients on longer regimens (Strong recommendation, moderate certainty in the estimates of effect) (No change)
Bedaquiline should be included in longer MDR-TB regimens for patients aged ≥18 years (Strong recommendation, moderate certainty in the estimates of effect) Bedaquiline may also be included in longer MDR-TB regimens for patients aged 6–17 years (Conditional recommendation, very low certainty in the estimates of effect)	3.4 Bedaquiline should be included in longer MDR-TB regimens for patients aged ≥18 years (Strong recommendation, moderate certainty in the estimates of effect) Bedaquiline may also be included in longer MDR-TB regimens for patients aged 6–17 years (Conditional recommendation, very low certainty in the estimates of effect) (No change)
Linezolid should be included in the treatment of MDR/RR-TB patients on longer regimens (Strong recommendation, moderate certainty in the estimates of effect)	3.5 Linezolid should be included in the treatment of MDR/RR-TB patients on longer regimens (Strong recommendation, moderate certainty in the estimates of effect) (No change)
Clofazimine and cycloserine or terizidone may be included in the treatment of MDR/RR-TB patients on longer regimens (Conditional recommendation, very low certainty in the estimates of effect)	3.6 Clofazimine and cycloserine or terizidone may be included in the treatment of MDR/RR-TB patients on longer regimens (Conditional recommendation, very low certainty in the estimates of effect) (No change)
Ethambutol may be included in the treatment of MDR/RR-TB patients on longer regimens (Conditional recommendation, very low certainty in the estimates of effect)	3.7 Ethambutol may be included in the treatment of MDR/RR-TB patients on longer regimens (Conditional recommendation, very low certainty in the estimates of effect) (No change)
Delamanid may be included in the treatment of MDR/RR-TB patients aged ≥3 years on longer regimens (Conditional recommendation, moderate certainty in the estimates of effect)	3.8 Delamanid may be included in the treatment of MDR/RR-TB patients aged ≥3 years on longer regimens (Conditional recommendation, moderate certainty in the estimates of effect) (No change)
Pyrazinamide may be included in the treatment of MDR/RR-TB patients on longer regimens (Conditional recommendation, very low certainty in the estimates of effect)	3.9 Pyrazinamide may be included in the treatment of MDR/RR-TB patients on longer regimens (Conditional recommendation, very low certainty in the estimates of effect) (No change)
Imipenem–cilastatin or meropenem may be included in the treatment of MDR/RR-TB patients on longer regimens (Conditional recommendation, very low certainty in the estimates of effect)	3.10 Imipenem–cilastatin or meropenem may be included in the treatment of MDR/RR-TB patients on longer regimens (Conditional recommendation, very low certainty in the estimates of effect)^ƒƒ^ (No change)
Amikacin may be included in the treatment of MDR/RR-TB patients aged ≥18 years on longer regimens when susceptibility has been demonstrated and adequate measures to monitor for adverse reactions can be ensured. If amikacin is not available, streptomycin may replace amikacin under the same conditions (Conditional recommendation, very low certainty in the estimates of effect)	3.11 Amikacin may be included in the treatment of MDR/RR-TB patients aged ≥18 years on longer regimens when susceptibility has been demonstrated and adequate measures to monitor for adverse reactions can be ensured. If amikacin is not available, streptomycin may replace amikacin under the same conditions (Conditional recommendation, very low certainty in the estimates of effect) (No change)
Ethionamide or prothionamide may be included in the treatment of MDR/RR-TB patients on longer regimens only if bedaquiline, linezolid, clofazimine or delamanid are not used or if better options to compose a regimen are not possible (Conditional recommendation against use, very low certainty in the estimates of effect)	3.12 Ethionamide or prothionamide may be included in the treatment of MDR/RR-TB patients on longer regimens only if bedaquiline, linezolid, clofazimine or delamanid are not used, or if better options to compose a regimen are not possible (Conditional recommendation against use, very low certainty in the estimates of effect) (No change)
p-Aminosalicylic acid may be included in the treatment of MDR/RR-TB patients on longer regimens only if bedaquiline, linezolid, clofazimine or delamanid are not used or if better options to compose a regimen are not possible (Conditional recommendation against use, very low certainty in the estimates of effect)	3.13 p-Aminosalicylic acid may be included in the treatment of MDR/RR-TB patients on longer regimens only if bedaquiline, linezolid, clofazimine or delamanid are not used, or if better options to compose a regimen are not possible (Conditional recommendation against use, very low certainty in the estimates of effect) (No change)
Clavulanic acid should not be included in the treatment of MDR/RR-TB patients on longer regimens (Strong recommendation against use, low certainty in the estimates of effect)^¶^	3.14 Clavulanic acid should not be included in the treatment of MDR/RR-TB patients on longer regimens (Strong recommendation against use, low certainty in the estimates of effect)^ƒƒ^ (No change)
**Section 3: The duration of longer MDR-TB regimens**	**Section 3: Longer regimens for MDR/RR-TB**
In MDR/RR-TB patients on longer regimens, a total treatment duration of 18–20 months is suggested for most patients; the duration may be modified according to the patient's response to therapy (Conditional recommendation, very low certainty in the estimates of effect)	3.15 In MDR/RR-TB patients on longer regimens, a total treatment duration of 18–20 months is suggested for most patients; the duration may be modified according to the patient's response to therapy (Conditional recommendation, very low certainty in the estimates of effect) (No change to wording, but combined with section above: Section 3: Recommendations on the use of longer regimens for MDR/RR-TB)
In MDR/RR-TB patients on longer regimens, a treatment duration of 15–17 months after culture conversion is suggested for most patients; the duration may be modified according to the patient's response to therapy (Conditional recommendation, very low certainty in the estimates of effect)	3.16 In MDR/RR-TB patients on longer regimens, a treatment duration of 15–17 months after culture conversion is suggested for most patients; the duration may be modified according to the patient's response to therapy (Conditional recommendation, very low certainty in the estimates of effect) (No change to wording, but combined with section above: Section 3: Recommendations on the use of longer regimens for MDR/RR-TB)
In MDR/RR-TB patients on longer regimens that contain amikacin or streptomycin, an intensive phase of 6–7 months is suggested for most patients; the duration may be modified according to the patient's response to therapy (Conditional recommendation, very low certainty in the estimates of effect)	3.17 In MDR/RR-TB patients on longer regimens containing amikacin or streptomycin, an intensive phase of 6–7 months is suggested for most patients; the duration may be modified according to the patient's response to therapy (Conditional recommendation, very low certainty in the estimates of effect) (No change to wording, but combined with section above: Section 2.2: Recommendations on the use of longer regimens for MDR/RR-TB)
**Section 4: Use of the standardised shorter MDR-TB regimen**	**Section 2: Shorter, all-oral, bedaquiline-containing regimen for MDR/RR-TB**
In MDR/RR-TB patients who have not been previously treated for >1 month with second-line medicines used in the shorter MDR-TB regimen or in whom resistance to fluoroquinolones and second-line injectable agents has been excluded, a shorter MDR-TB regimen of 9–12 months may be used instead of the longer regimens (Conditional recommendation, low certainty in the estimates of effect)	2.1 A shorter all-oral bedaquiline-containing regimen of 9–12 months duration is recommended in eligible patients with confirmed MDR/RR-TB who have not been exposed to treatment with second-line TB medicines used in this regimen for >1 month, and in whom resistance to fluoroquinolones has been excluded (Conditional recommendation, very low certainty in the evidence) (Updated recommendation)
Not included in 2019 guidelines	**Section 4: The BPaL regimen for MDR-TB with additional fluoroquinolone resistance**
Not included in 2019 guidelines	4.1 A treatment regimen lasting 6–9 months, composed of bedaquiline, pretomanid and linezolid (BPaL), may be used under operational research conditions in MDR-TB patients with TB that is resistant to fluoroquinolones, who have either had no previous exposure to bedaquiline and linezolid or have been exposed for ≤2 weeks (Conditional recommendation, very low certainty in the estimates of effect) (New recommendation)
**Section 5: Monitoring patient response to MDR-TB treatment using culture**	**Section 5: Monitoring patient response to MDR-TB treatment using culture**
In MDR/RR-TB patients on longer regimens, the performance of sputum culture in addition to sputum smear microscopy is recommended to monitor treatment response. It is desirable for sputum culture to be repeated at monthly intervals (Strong recommendation, moderate certainty in the estimates of test accuracy)	5.1 In MDR/RR-TB patients on longer regimens, the performance of sputum culture in addition to sputum smear microscopy is recommended to monitor treatment response. It is desirable for sputum culture to be repeated at monthly intervals (Strong recommendation, moderate certainty in the estimates of test accuracy) (No change)
**Section 6: Start of antiretroviral therapy in patients on second-line anti-TB regimens**	**Section 6: Start of antiretroviral therapy in patients on second-line anti-TB regimens**
Antiretroviral therapy is recommended for all patients with HIV and drug-resistant TB requiring second-line anti-TB drugs, irrespective of CD4 cell count, as early as possible (within the first 8 weeks) following initiation of anti-TB treatment (Strong recommendation, very low-quality evidence)	6.1 Antiretroviral therapy is recommended for all patients with HIV and drug-resistant TB requiring second-line anti-TB drugs, irrespective of CD4 cell count, as early as possible (within the first 8 weeks) following initiation of anti-TB treatment (Strong recommendation, very low quality evidence) (No change)
**Section 7: Surgery for patients on MDR-TB treatment**	**Section 7: Surgery for patients on MDR-TB treatment**
In patients with RR-TB or MDR-TB, elective partial lung resection (lobectomy or wedge resection) may be used alongside a recommended MDR-TB regimen (Conditional recommendation, very low certainty in the evidence)	7.1 In patients with RR-TB or MDR-TB, elective partial lung resection (lobectomy or wedge resection) may be used alongside a recommended MDR-TB regimen (Conditional recommendation, very low certainty in the evidence) (No change)
**Section 8: Care and support for patients with MDR/RR-TB**	**Section 8: Care and support for patients with MDR/RR-TB**
Health education and counselling on the disease and treatment adherence should be provided to patients on TB treatment (Strong recommendation, moderate certainty in the evidence)	8.1 Health education and counselling on the disease and treatment adherence should be provided to patients on TB treatment (Strong recommendation, moderate certainty in the evidence) (No change)
A package of treatment adherence interventions^+^ may be offered to patients on TB treatment in conjunction with the selection of a suitable treatment administration option (Conditional recommendation, low certainty in the evidence)^§^	8.2 A package of treatment adherence interventions^+^ may be offered to patients on TB treatment in conjunction with the selection of a suitable treatment administration option^§^ (No change)
One or more of the following treatment adherence interventions (complementary and not mutually exclusive) may be offered to patients on TB treatment or to healthcare providers:	8.3 One or more of the following treatment adherence interventions (complementary and not mutually exclusive) may be offered to patients on TB treatment or to healthcare providers:
1) tracers^ƒ^ and/or digital medication monitor^##^ (conditional recommendation, very low certainty in the evidence)	a) tracers^ƒ^ and/or digital medication monitor^##^ (conditional recommendation, very low certainty in the evidence)
2) material support^¶¶^ to the patient (conditional recommendation, moderate certainty in the evidence)	b) material support^¶¶^ to the patient (conditional recommendation, moderate certainty in the evidence)
3) psychological support^++^ to the patient (conditional recommendation, low certainty in the evidence)	c) psychological support^++^ to the patient (conditional recommendation, low certainty in the evidence)
4) staff education^§§^ (conditional recommendation, low certainty in the evidence)	d) staff education^§§^ (conditional recommendation, low certainty in the evidence) (No change)
The following treatment administration options may be offered to patients on TB treatment:	8.4 The following treatment administration options may be offered to patients on TB treatment:
a) community- or home-based DOT is recommended over health facility-based DOT or unsupervised treatment (conditional recommendation, moderate certainty in the evidence)	a) community- or home-based DOT is recommended over health facility-based DOT or unsupervised treatment (conditional recommendation, moderate certainty in the evidence)
b) DOT administered by trained lay providers or healthcare workers is recommended over DOT administered by family members or unsupervised treatment (conditional recommendation, very low certainty in the evidence)	b) DOT administered by trained lay providers or healthcare workers is recommended over DOT administered by family members or unsupervised treatment (conditional recommendation, very low certainty in the evidence)
c) VOT may replace DOT when video communication technology is available, and it can be appropriately organised and operated by healthcare providers and patients (conditional recommendation, very low certainty in the evidence)	c) VOT may replace DOT when the video communication technology is available, and it can be appropriately organised and operated by healthcare providers and patients (conditional recommendation, very low certainty in the evidence)
	(No change)
Patients with MDR-TB should be treated using mainly ambulatory care rather than models of care based principally on hospitalisation (Conditional recommendation, very low-quality evidence)	8.5 Patients with MDR-TB should be treated using mainly ambulatory care rather than models of care based principally on hospitalisation (Conditional recommendation, very low quality evidence) (No change)
A decentralised model of care is recommended over a centralised model for patients on MDR-TB treatment (Conditional recommendation, very low certainty in the evidence)	8.6 A decentralised model of care is recommended over a centralised model for patients on MDR-TB treatment (Conditional recommendation, very low certainty in the evidence) (No change)

The *WHO Consolidated Guidelines on Tuberculosis, Module 4: Drug-Resistant Tuberculosis Treatment* were a compilation of existing and new recommendations on the treatment and management of MDR/RR-TB and as such they included new recommendations published in 2019 and existing recommendations that had been previously published. In the current update (2020), there are two new recommendations (recommendations 2.1 and 4.1) and a minor change to the wording of a pre-existing recommendation (recommendation 3.1). Recommendation 2.1 is an update to a previous recommendation on shorter regimens for MDR/RR-TB while recommendation 4.1 was based on a new Population, Intervention, Comparator, Outcomes (PICO) question concerning the bedaquiline, pretomanid and linezolid (BPaL) regimen. Recommendations on the duration of longer regimens for MDR/RR-TB (recommendations 3.15, 3.16 and 3.17) were combined into the section on the composition of longer regimens for MDR/RR-TB (recommendations 3.1–3.14); however, the wording of the recommendations on duration remained unchanged. All other recommendations remain unchanged. DOT: directly observed treatment; VOT: video-observed treatment. ^#^: group A = levofloxacin/moxifloxacin, bedaquiline, linezolid; group B = clofazimine, cycloserine/terizidone; group C = ethambutol, delamanid, pyrazinamide, imipenem–cilastatin, meropenem, amikacin (streptomycin), ethionamide/prothionamide, p-aminosalicylic acid (see also table 3). ^¶^: imipenem–cilastatin and meropenem are administered with clavulanic acid, which is available only in formulations combined with amoxicillin (amoxicillin–clavulanic acid). When included, clavulanic acid is not counted as an additional effective TB agent and should not be used without imipenem–cilastatin or meropenem. ^+^: treatment adherence interventions include social support such as material support (*e.g*. food, financial incentives, transport fees), psychological support, tracers such as home visits or digital health communications (*e.g*. short message service (SMS), telephone calls), medication monitor and staff education. The interventions should be selected based on an assessment of the individual patient's needs, provider's resources and conditions for implementation. ^§^: treatment administration options include DOT, non-daily DOT, VOT or unsupervised treatment. ^ƒ^: tracers refer to communication with the patient, including home visits or *via* SMS, telephone (voice) call. ^##^: a digital medication monitor is a device that can measure the time between openings of the pill box. The medication monitor can have audio reminders or send an SMS to remind the patient to take medications, along with recording when the pill box is opened. ^¶¶^: material support can be food or financial support: meals, food baskets, food supplements, food vouchers, transport subsidies, living allowance, housing incentives or financial bonus. This support addresses the indirect costs incurred by patients or their attendants in order to access health services and, possibly, tries to mitigate the consequences of income loss related to the disease. ^++^: psychological support can be counselling sessions or peer-group support. ^§§^: staff education can be adherence education, chart or visual reminders, educational tools and desktop aids for decision-making and reminders. ^ƒƒ^: imipenem–cilastatin and meropenem are administered with clavulanic acid, which is available only in formulations combined with amoxicillin. Amoxicillin–clavulanic acid is not counted as an additional effective TB agent, and should not be used without imipenem–cilastatin or meropenem.

## Discussion

The 2020 revisions to the WHO treatment guidelines for drug-resistant TB signal important changes for national TB programmes and other organisations that are involved in the treatment of patients with drug-resistant TB, worldwide. The standardised all-oral bedaquiline-containing shorter regimen is the preferred treatment regimen for eligible patients. Some patient subgroups such as pregnant women with TB (for whom ethionamide is contraindicated), patients with previous exposure to the medicines used in the regimen or patients with extensive TB disease or severe extrapulmonary TB are not eligible for the shorter regimen and will need to start an all-oral longer regimen composed using the hierarchy of recommended medicines in groups A, B and C. Similarly, patients who have MDR/RR-TB and fluoroquinolone resistance, which globally represent one-fifth of all those with MDR/RR-TB will require a longer regimen unless they are enrolled in an operational research study where the BPaL regimen is being offered. The very low certainty in the evidence for the two new recommendations reinforces the importance of future research on MDR-TB treatment regimens, not only for BPaL, but also for other regimens, including any modifications to the recommended shorter regimen.

These recommendations reinforce the importance of several aspects of national TB programming, which need to be well functioning in order to diagnose and treat MDR/RR-TB effectively. Among these are the availability of quality assured diagnostics, drug DST, acquisition of drug resistance, cross-resistance, active drug safety monitoring and management (aDSM) and social support to patients [16, 45]. Currently, to diagnose MDR/RR-TB, the WHO recommends rapid molecular tests as the initial test to diagnose pulmonary TB and to simultaneously detect rifampicin resistance [46]. The most recent WHO guidance also supports the use of molecular diagnostics in the diagnostic work-up of extrapulmonary TB and in children with TB (specifically in gastric specimens, nasopharyngeal specimens and stool specimens) [46]. In addition, WHO recommends that for patients with confirmed MDR/RR-TB, second-line probe assay may be used as the initial test, instead of phenotypic culture-based DST, to detect resistance to fluoroquinolones [47]. Testing of fluoroquinolone resistance will be essential for the roll out of the shorter regimen, as it contains a fluoroquinolone, and preventing resistance to other medicines in the regimen is paramount. In addition, DST is important to ensure that the longer regimen is composed of the most effective medicines for that particular patient, as well as preventing amplification of resistance to other effective new anti-TB medicines. It is vital that patients have access to reliable DST for medicines that are in use and countries include those medicines in their surveillance schemes, and eventually it would be desirable for laboratories to be able to carry out rapid DST for medicines such as bedaquiline and linezolid, which are likely to feature more prominently in MDR-TB treatment regimens from now on. Countries that have insufficient laboratory capacity to perform DST (especially for the fluoroquinolones) should aim to strengthen this capacity as a priority. Despite the fact that DST for some of the medicines in the newly recommended regimens needs further strengthening, a WHO technical consultation in 2017 established critical concentrations for susceptibility testing for the fluoroquinolones, bedaquiline, delamanid, clofazimine and linezolid [48]. More advanced laboratory methods such as next-generation or whole-genome sequencing may be available in some countries, but are not widely available to all national TB programmes globally. However, national data on the prevalence of resistance to certain medicines, including through targeted or whole-genome sequencing, could help guide policies on treatment. Preventing the acquisition of such drug resistance is also paramount, as is the implementation of aDSM [49].

**FIGURE 1:**
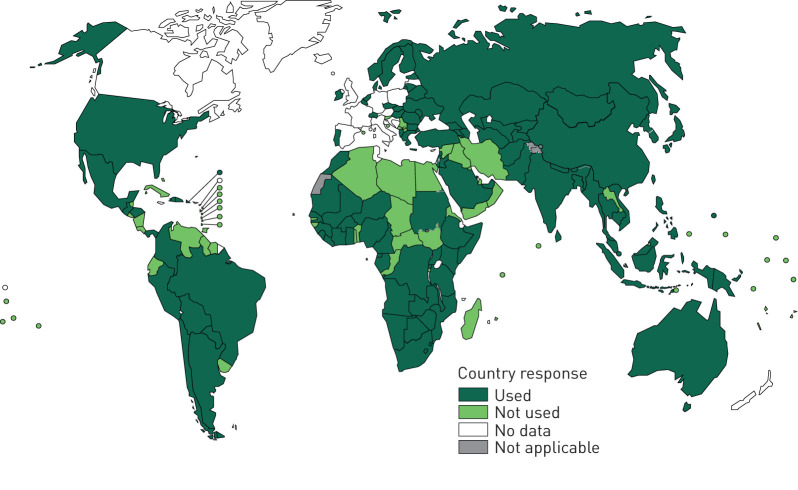
Countries that used bedaquiline for the treatment of multidrug-resistant/extensively drug-resistant tuberculosis as part of expanded access, compassionate use or under normal programmatic conditions by the end of 2019.

aDSM is recommended in the context of the introduction of new anti-TB drugs and novel MDR-TB regimens into national TB programmes. The overall objective is “to reduce risks from drug-related harms in patients on second-line treatment for drug-resistant TB and to generate standardised aDSM data to inform future policy updates on the use of such medicines” [49]. Greater adoption of aDSM is encouraged as shorter and longer regimens now contain bedaquiline [50]. Based on the latest data available to the WHO (reported by 186 countries in 2020), 109 countries (59% of those who reported) had used bedaquiline for the treatment of MDR/XDR-TB as part of expanded access, compassionate use or under normal programmatic conditions by the end of 2019 (figure 1). Patient support has been a feature of several shorter and longer regimens and continues to be an important component of MDR-TB treatment. Recommendations on patient social support are also part of the new WHO guidelines [7].

The research gaps identified by the GDG in November 2019 reflect current gaps in knowledge about the shorter regimens and the BPaL regimen (table 6) [7]. Additional research is needed on the effectiveness and safety of variants of the shorter MDR-TB treatment regimen and where the total duration of treatment is reduced to ≤6 months. The South African national TB programme has recently further modified the shorter regimen to include linezolid instead of ethionamide [51] and the results of this modified regimen and others under research will probably help to inform policy in the future. In addition, comparison of the effectiveness of these variants of the shorter regimen would be helpful in specific patient subgroups that have been excluded from previous studies, such as children, patients with additional resistance, those with extrapulmonary TB or extensive TB disease, pregnant and breastfeeding women or in settings where background resistance to drugs other than fluoroquinolones and second-line injectable agents is high (*e.g.* pyrazinamide or high-level isoniazid resistance). Overall, there is a continued need for additional high-quality research, including operational research and randomised controlled trials, that allows the comparison of all-oral shorter regimens to all-oral longer regimens, and provides more data on the use of all-oral shorter regimens in settings other than South Africa. The frequency and mechanisms of bedaquiline resistance acquisition are another important research priority. For the BPaL regimen the current recommendation is conditional upon its implementation under operational research conditions. Therefore, additional research on the implementation of the BPaL regimen will be required from other regions and countries beyond South Africa. As pretomanid is a new compound [52], it will be important to describe the mechanism and molecular markers of pretomanid resistance, and to document its full adverse event profile with a focus on hepatotoxicity and reproductive toxicity in humans. More data on post-treatment follow-up is expected to be available and is important to understand efficacy of shorter treatment regimens. In addition, in the Nix-TB study, almost one-third of patients stopped linezolid early due to an adverse event, underscoring the need for additional research on the optimal dose and duration of linezolid for use in drug-resistant TB regimens [39]. The ZeNix study is currently addressing these questions and it will provide important new information to help guide linezolid dosing in the future (clinicaltrials.gov identifier number NCT03086486). A summary of research priorities identified by the GDG is included in table 5.

**TABLE 6:** Research priorities on treatment regimens for drug-resistant tuberculosis (TB)

**Shorter all-oral bedaquiline-containing regimen for MDR/RR-TB**
The effectiveness and safety of variants of the shorter MDR-TB treatment regimen, in which the injectable agent is replaced by an oral agent (*e.g.* bedaquiline) and the total duration is reduced to ≤6 months Comparison of the effectiveness of these variants of the shorter regimen would be helpful in:
• patient subgroups that have often been systematically excluded from studies or country programme cohorts (*e.g.* children, patients with additional resistance, those with extrapulmonary TB and pregnant or breastfeeding women)
• settings where background resistance to drugs other than fluoroquinolones and second-line injectable agents is high (*e.g.* pyrazinamide or high-level isoniazid resistance)
Additional RCTs and odds ratios on all-oral shorter MDR-TB treatment regimens, also allowing comparison of all-oral shorter regimens to all-oral longer regimens Programmatic data from countries other than South Africa Data from children, pregnant women, the elderly, patients with diabetes and other special populations Data on patients presenting with extensive TB disease Information on the frequency and mechanisms of bedaquiline resistance acquisition, and the genetic markers that indicate likely resistance Identification of optimal companion drugs that protect bedaquiline and limit the acquisition of bedaquiline resistance, including consideration of the need to protect the long “tail” of potential single drug exposure (given its exceptionally long half-life) if bedaquiline is stopped at the same time as companion drugs
**Longer regimens for MDR/RR-TB**
The optimal combination of medicines and approach to regimen design for adults and children with MDR/RR-TB, with or without additional resistance to key agents RCTs, which there is a lack of, especially those involving new drugs and regimens: the release of results from the first phase III trials for MDR-TB has led to debate about the clinical relevance of the design and end-points chosen for these studies, requiring at times additional off-protocol analysis of data to explore the potential added value of the experimental interventions Inclusion and separate reporting of outcomes for key subgroups in RCTs, especially children, pregnant and breastfeeding women and HIV-positive individuals on treatment Studies of pharmacokinetics and safety to determine optimal drug dosing (especially in pregnancy), and the effect of extemporaneous manipulation of existing dosing forms Complete recording of adverse events and standardised data on organ class, seriousness, severity and certainty of association to allow meaningful comparison of the association between adverse events and exposure to different medicines between studies, patient subgroups and different regimens Determination of the minimum number of drugs and treatment duration (especially in patients previously treated for MDR-TB) Improved diagnostics and DST methods (*e.g.* which test to use for resistance to pyrazinamide) especially for medicines for which no rapid molecular methods are currently available in the field Further research and development would be particularly helpful for the following agents:
• levofloxacin: optimisation of the dose: the Opti-Q study will soon provide new information on this (clinicaltrials.gov identifier number NCT01918397)
• bedaquiline: use in children to determine optimal pharmacokinetic properties, revised cost–effectiveness analyses based on the IPD meta-analysis, optimisation of duration in both adults and children, and use during pregnancy
• linezolid: optimisation of the dose and duration in both adults and children, and patient predictors for adverse reactions
• clofazimine: optimisation of the dose, especially in children, any added value in using a loading dose and availability of DST methods
• cycloserine and terizidone: differences in efficacy between the two medicines, approaches to test for susceptibility to them, and best practices in psychiatric care for people on these medicines
• delamanid: better understanding of its role in MDR-TB regimens, including in children (pharmacokinetics/pharmacodynamics), pregnant women and people living with HIV; mechanisms of development of drug resistance; and optimisation of duration in both adults and children
• pyrazinamide: molecular testing for resistance (pursuing either LPA or other approaches)
• carbapenems: given their effectiveness in the evidence reviews, further research on their role in MDR-TB regimens is important, including the potential role and cost-effectiveness of ertapenem (which can be given intramuscularly) as a substitute for meropenem and imipenem–cilastatin
• amikacin: the safety and effectiveness of thrice-weekly administration at a higher dose (∼25 mg·kg^−1^ per day) [56]
Identification of factors that determine the optimal duration of treatment (*e.g.* previous treatment history, baseline resistance patterns, site of disease and age) Exploration of strategies to optimise the balance of benefits *versus* harms of regimen duration through risk-stratification approaches
**The BPaL regimen for MDR-TB with additional fluoroquinolone resistance**
The efficacy, safety and tolerability of BPaL compared with other all-oral regimens Description of the mechanism and molecular markers of pretomanid resistance, and surveillance for the development of resistance with adequate consideration paid to the impact of selected mutations
Data from other regions and countries (beyond South Africa)
Documenting the full adverse event profile of pretomanid, and the frequency of relevant adverse events, with a focus on hepatotoxicity and reproductive toxicity in humans (the reproductive toxicities of pretomanid have been signalled in animal studies, but the potential effects of this medicine on human fertility have not been adequately evaluated) Exploring the relative efficacy (and added value in multidrug regimens) of pretomanid and delamanid Optimal dose and duration of linezolid use in drug-resistant TB regimens (ZeNix study)

MDR: multidrug-resistant; RR: rifampin-resistant; BPaL: bedaquiline, pretomanid and linezolid; RCT: randomised controlled trial; IPD: individual patient dataset; DST: drug susceptibility testing; LPA: line probe assay.

To promote further research on treatment regimens for MDR/RR-TB, the Special Programme for Research and Training in Tropical Diseases and the WHO have launched ShORRT (Short, all-Oral Regimens for Rifampicin-resistant Tuberculosis), an operational research package to assess the effectiveness, safety, feasibility, acceptability, cost and impact (including on health-related quality of life) of the use of all-oral shorter treatment regimens for adults and children with MDR/RR-TB [53]. The package promotes research on shorter regimens for MDR-TB patients with and without fluoroquinolone resistance, including an all-oral shorter regimen which contains bedaquiline and linezolid and the BPaL regimen [53].

Other issues regarding implementation of these new regimens will be important for national TB programmes and other stakeholders involved in the programmatic management of drug-resistant TB. National TB programmes should plan to transition to the new regimens as soon as possible, removing the need for the injectable agents; they should also strive to improve equitable access to newer medicines such as bedaquiline. Countries who receive funding for their national TB programmes through the Global Fund will be able to incorporate these newer regimens into funding applications and there are advocacy efforts underway to reduce the costs of certain medicines such as bedaquiline, which now features in both shorter and longer regimens and in the BPaL regimen. Stakeholders should also be aware that further changes to regimens may be expected in the future as new evidence becomes available.

In addition, while the recommendations are mainly concordant when comparing WHO guidelines on the treatment of drug resistant TB to the clinical practice guideline issued by the American Thoracic Society (ATS)/Centers for Disease Control and Prevention (CDC)/European Respiratory Society (ERS)/Infectious Diseases Society of America (IDSA), there are also some differences [54]. These differences are not significant and are summarised in full in the clinical practice guideline, therefore they are not replicated here [54]. While similar methods, evidence and the application of GRADE were used for both guidelines, the differences can be explained by the fact that the WHO guidelines were based on a modified dataset that expanded on the dataset used to inform the ATS/CDC/ERS/IDSA clinical practice guideline, including a comparatively large dataset from South Africa [54]. The datasets used for both guidelines overlapped substantially, but were not exactly the same [54]. In addition, the intended audience for WHO guidelines is global and caters for settings that differ in prevalence, resources and health infrastructure when compared to ATS/CDC/ERS/IDSA and other guidelines.

### Limitations

WHO recommendations are based on the latest evidence available, using analyses that aim to limit the effect of confounding and in the context of the GRADE process; however, limitations remain. Both of the new recommendations on the shorter regimen and the BPaL regimen are conditional. Conditional recommendations have different implications from the perspectives of patients, clinicians and policy-makers (table 4). Conditional recommendations are usually made when the desirable effects outweigh the undesirable effects but some uncertainty exists based on the judgements made in the EtD framework [55]. In addition, both recommendations are based on very low certainty evidence, which is assessed based on a number of domains including inconsistency, indirectness and imprecision [55]. This highlights the need for additional high-quality evidence on treatment regimens for drug-resistant TB, including from trials and observational data. The GRADE tables which describe the quality of the evidence and the EtD frameworks, which outline the judgements made to arrive at recommendations are included in the guideline annexes [37]. Furthermore, while all efforts are made to limit the effect of confounding in the analyses of data used to inform policy-making, it is possible that residual confounding exists. In fact, this was one reason why recommendations were not possible for two PICO questions during the GDG meeting held in 2019.

### Conclusions

The 2020 updates to the WHO drug-resistant TB treatment guidelines provide new recommendations on effective regimens for the treatment of MDR/RR-TB based on the latest evidence available to the WHO [7]. National TB programmes and other stakeholders involved in the treatment of MDR/RR-TB should align national policies with these recommendations, while also pursuing the vital research that is needed to address the identified research priorities. Adoption of new and existing recommendations along with their safe and timely implementation, in the context of patient support, will require political commitment with the involvement of national governments, the private sector, technical partners, civil society and funding agencies. Further high-quality research, including operational research and randomised controlled trials, continues to be urgently needed to improve treatment outcomes and quality of life for patients with drug-resistant TB worldwide.

## Shareable PDF

10.1183/13993003.03300-2020.Shareable1 This one-page PDF can be shared freely online.
Shareable PDF ERJ-03300-2020.Shareable
