# Supplementary material for: World Health Organization recommendations on the treatment of drug-resistant tuberculosis, 2020 update
Source: Eur Respir J. 2021 Jun 3;57(6):2003300. doi: 10.1183/13993003.03300-2020 (PMC8176349; doi:10.1183/13993003.03300-2020)
Supplement: Supplementary file 1 [file ERJ-03300-2020.Shareable.pdf]

SHAREABLE PDF

World Health Organization recommendations on the treatment of drug-resistant tuberculosis, 2020 update

Fuad Mirzayev, Kerri Viney, Nguyen Nhat Linh, Licé Gonzalez-Angulo, Medea Gegia, Ernesto Jaramillo, Matteo Zignol and Tereza Kasaeva

Affiliation: Global TB Programme, World Health Organization, Geneva, Switzerland.

Correspondence: Fuad Mirzayev, Global TB Programme, World Health Organization, Avenue Appia 20, 1211 Geneva, Switzerland. E-mail: mirzayevf@who.int

@ERSpublications
New WHO guidelines on the treatment of drug-resistant tuberculosis (TB) contain the latest recommendations on shorter or longer all oral treatment regimens for patients with drug-resistant TB, including the medicines to be used and other supportive measures https://bit.ly/2UJeib7

Cite this article as: Mirzayev F, Viney K, Linh NN, *et al*. World Health Organization recommendations on the treatment of drug-resistant tuberculosis, 2020 update. *Eur Respir J* 2021; 57: 2003300 [https://doi.org/10.1183/13993003.03300-2020].

This single-page version can be shared freely online.

ABSTRACT Antimicrobial resistance is a major public health problem globally. Likewise, forms of tuberculosis (TB) resistant to first- and second-line TB medicines present a major challenge for patients, healthcare workers and healthcare services. In November 2019, the World Health Organization (WHO) convened an independent international expert panel to review new evidence on the treatment of multidrug- (MDR) and rifampicin-resistant (RR) TB, using the Grading of Recommendations Assessment, Development and Evaluation approach.

Updated WHO guidelines emerging from this review, published in June 2020, recommend a shorter treatment regimen for patients with MDR/RR-TB not resistant to fluoroquinolones (of 9–11 months), with the inclusion of bedaquiline instead of an injectable agent, making the regimen all oral. For patients with MDR-TB and additional fluoroquinolone resistance, a regimen composed of bedaquiline, pretomanid and linezolid may be used under operational research conditions (6–9 months). Depending on the drug-resistance profile, extent of TB disease or disease severity, a longer (18–20 months) all-oral, individualised treatment regimen may be used. In addition, the review of new data in 2019 allowed the WHO to conclude that there are no major safety concerns on the use of bedaquiline for >6 months' duration, the use of delamanid and bedaquiline together and the use of bedaquiline during pregnancy, although formal recommendations were not made on these topics.

The 2020 revision has highlighted the ongoing need for high-quality evidence and has reiterated the need for clinical trials and other research studies to contribute to the development of evidence-based policy.

Copyright ©The authors 2021. This version is distributed under the terms of the Creative Commons Attribution Licence 4.0.
